# Longitudinal optical coherence tomography to visualize the in vivo response of middle ear biofilms to antibiotic therapy

**DOI:** 10.1038/s41598-021-84543-9

**Published:** 2021-03-04

**Authors:** Jungeun Won, Wenzhou Hong, Pawjai Khampang, Darold R. Spillman, Samuels Marshall, Ke Yan, Ryan G. Porter, Michael A. Novak, Joseph E. Kerschner, Stephen A. Boppart

**Affiliations:** 1 Department of Bioengineering, University of Illinois at Urbana-Champaign, Urbana, IL USA; 2 Beckman Institute for Advanced Science and Technology, University of Illinois at Urbana-Champaign, Urbana, IL USA; 3 Department of Otolaryngology and Communication Sciences, Medical College of Wisconsin, Milwaukee, WI USA; 4 Section of Quantitative Health Sciences, Department of Pediatrics, Medical College of Wisconsin, Milwaukee, WI USA; 5 Department of Otolaryngology, Carle Foundation Hospital, Urbana, IL USA; 6 Carle Illinois College of Medicine, University of Illinois at Urbana-Champaign, Champaign, IL USA; 7 Division of Otolaryngology and Pediatric Otolaryngology, Medical College of Wisconsin, Milwaukee, WI USA; 8 Department of Electrical and Computer Engineering, University of Illinois at Urbana-Champaign, Urbana, IL USA

**Keywords:** Biomedical engineering, Imaging and sensing, Bacterial infection, Biofilms, Paediatrics

## Abstract

Studying the impact of antibiotic treatment on otitis media (OM), the leading cause of primary care office visits during childhood, is critical to develop appropriate treatment strategies. Tracking dynamic middle ear conditions during antibiotic treatment is not readily applicable in patients, due to the limited diagnostic techniques available to detect the smaller amount and variation of middle ear effusion (MEE) and middle ear bacterial biofilm, responsible for chronic and recurrent OM. To overcome these challenges, a handheld optical coherence tomography (OCT) system has been developed to monitor in vivo response of biofilms and MEEs in the OM-induced chinchilla model, the standard model for human OM. As a result, the formation of MEE as well as biofilm adherent to the tympanic membrane (TM) was longitudinally assessed as OM developed. Various types of MEEs and biofilms in the chinchilla model were identified, which showed comparable features as those in humans. Furthermore, the effect of antibiotics on the biofilm as well as the amount and type of MEEs was investigated with low-dose and high-dose treatment (ceftriaxone). The capability of OCT to non-invasively track and examine middle ear conditions is highly beneficial for therapeutic OM studies and will lead to improved management of OM in patients.

## Introduction

Otitis media (OM), also known as a middle ear infection, affects more than 80% of children in the United States^1^ and is one of the most common reasons for pediatrician visits and antibiotic prescription during childhood^2,3^. Acute OM (AOM)^4^ is caused by both viral and bacterial pathogens. For bacterial AOM, antibiotic therapy is often employed, however, antimicrobial resistance has decreased the efficacy of this intervention and increased the necessity for improved diagnostic techniques and novel interventions^5^.

Bacterial biofilms in the middle ear have been shown, at least in part, to contribute to decreased efficacy of antibiotics in treating OM^6,7^. A biofilm is a group of bacteria encased in an extracellular polymeric matrix, often adhered to a surface. Bacteria within the biofilm have a lower metabolic rate which, along with the encasing extracellular matrix, produces a greater resistance to antibiotics. The antibiotic resistance of bacteria within the biofilm structure have also been linked to chronic and recurrent OM^8^. Furthermore, studies have identified the presence of biofilm from children with chronic OM^9,10^ and recurrent acute OM (RAOM)^11^. Thus, it is critical to understand the effects of the antibiotic treatment on the biofilm, which may lead to better strategies to treat OM.

Middle ear effusion (MEE) is a hallmark of both acute and chronic OM. In AOM, progression of the MEE has the potential to inform the efficacy of treatment, such as antibiotic therapy. However, few studies have been performed in order to investigate the impact of antibiotic treatment on patients by monitoring the presence of a middle ear effusion (MEE)^12,13^. Difficulties associated with human studies in attempts to link MEE and treatment include a heterogeneous population related to severity and time course of the AOM, as well as other demographic factors including: previous infections, age of onset, overall immunologic status, and environment. In addition, follow-up intervals to longitudinally track the antibiotic therapy response in clinical settings can be long and inconsistent between patients. Finally, the current diagnostic techniques, such as standard otoscopy, pneumatic otoscopy, and tympanometry, have a limited capability to detect small volumes and variations of the MEE, and cannot detect the presence of biofilm. Each of these have presented difficulties in the design or interpretation of data in human studies.

Optical coherence tomography (OCT), developed in 1991^14^, is a non-invasive imaging technique that uses backscattered light to create cross-sectional depth-resolved images of tissue. OCT is analogous to ultrasound imaging, except reflections of near-infrared light are collected rather than sound, and the contrast in an OCT image is produced from differences in the refractive indices of the tissue, rather than differences in acoustic impedance. With a depth resolution of 2–10 µm, OCT is extensively used in ophthalmology for corneal and retinal imaging^15^, and has actively been investigated in many other applications, including cardiology^16^, oncology^17^, and dermatology^18^. In otolaryngology, several studies have demonstrated the capability of OCT in assessing the middle ear and the inner ear in human and animal models^6,19–22^. Furthermore, the presence of a biofilm as well as a MEE in the human middle ear can be non-invasively identified and assessed in vivo using OCT^10,23,24^. These studies have demonstrated great promise to improve current middle ear diagnostic technologies^25^.

Given the difficulties in human studies noted above, the ideal nature of the chinchilla animal model to study AOM^26–28^, and the inherent advantages of OCT in providing diagnostic information, the current investigation was initiated to provide a novel, robust, and reproducible dataset related to AOM, antibiotic therapy, biofilm formation, and resolution. An AOM-induced chinchilla model was employed to visualize the in vivo response of the MEE and biofilm to the antibiotic treatment. First, the formation of MEE and biofilm after inoculating with nontypeable *Haemophilus influenzae* (NTHi), one of the three primary bacteria strains causing OM in humans, was noninvasively and longitudinally observed using OCT. Next, different dosages of antibiotic treatments were administered to compare the effect of the treatment on the visualized MEEs and biofilms in OCT. The parameters, such as the thickness of the TM, the presence and thickness of any biofilm, and the presence and scattering properties of any MEE, were statistically assessed and compared with histology and fluorescence in situ hybridization (FISH). The capability of OCT in noninvasively imaging and longitudinally tracking the MEE and biofilm in chinchilla models will facilitate further understanding of the impact of antibiotic treatment in OM in humans, and can ultimately be used as a tool for OM therapeutic studies in patients.

## Results

### Imaging chinchilla middle ear cavity in vivo

**Figure 1:**
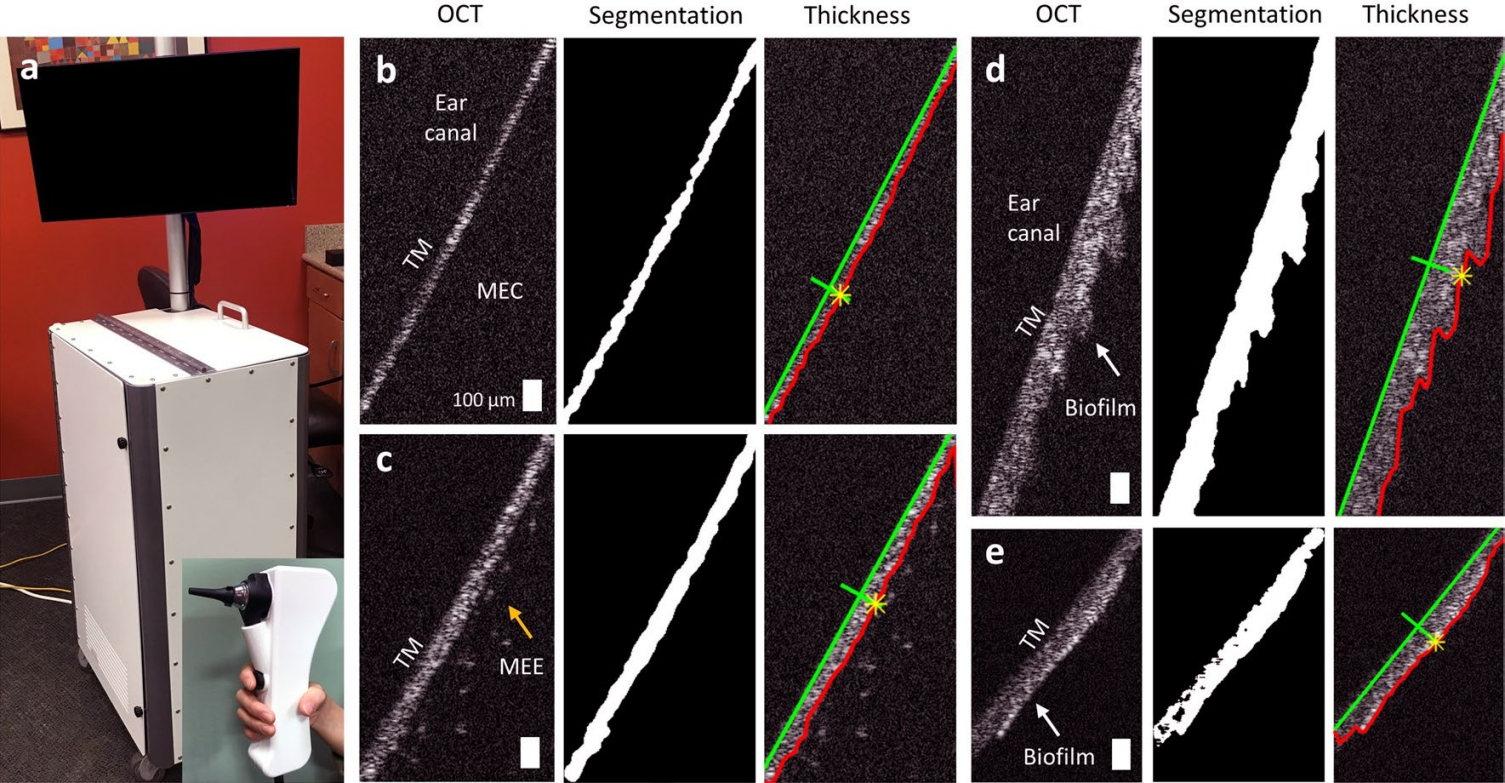
Handheld OCT system for middle ear imaging in the chinchilla model. (**a**) Photo of the OCT system and handheld probe (inset). Representative OCT images showing (**b**) clear middle ear cavity, (**c**) presence of a MEE, and (**d**,**e**) TM with a biofilm. The TM and any biofilm adherent to the TM was segmented. Next, the orthogonal distance from the green and the red line was measured and corrected for the refractive index (RI) error. Scale bars represent 100 µm. *TM* tympanic membrane, *MEC* middle ear cavity, *MEE* middle ear effusion.

A custom-built, portable, and handheld spectral-domain OCT system (Fig. 1a) was developed to non-invasively visualize the chinchilla middle ear (see the “Optical coherence tomography system” section in “Materials and methods”). A standard ear speculum was used for imaging. Video otoscopy (MedRx) was performed prior to OCT imaging to acquire high-resolution surface images of the chinchilla TM. The thickness of the TM was measured from OCT, as it can be correlated to the inflammation during OM^29^. Representative OCT images of a control chinchilla TM (Fig. 1b), a chinchilla TM with MEE (Fig. 1c), and biofilm (Fig. 1d,e), are illustrated in Fig. 1. Furthermore, the thickness of the TM, including any TM-adherent biofilm, was calculated at each spatial point on the image and corrected for the refractive index (RI) error (see the “OCT image analysis” section in “Materials and methods”).

### OCT monitoring of OM progression in chinchilla

**Figure 2:**
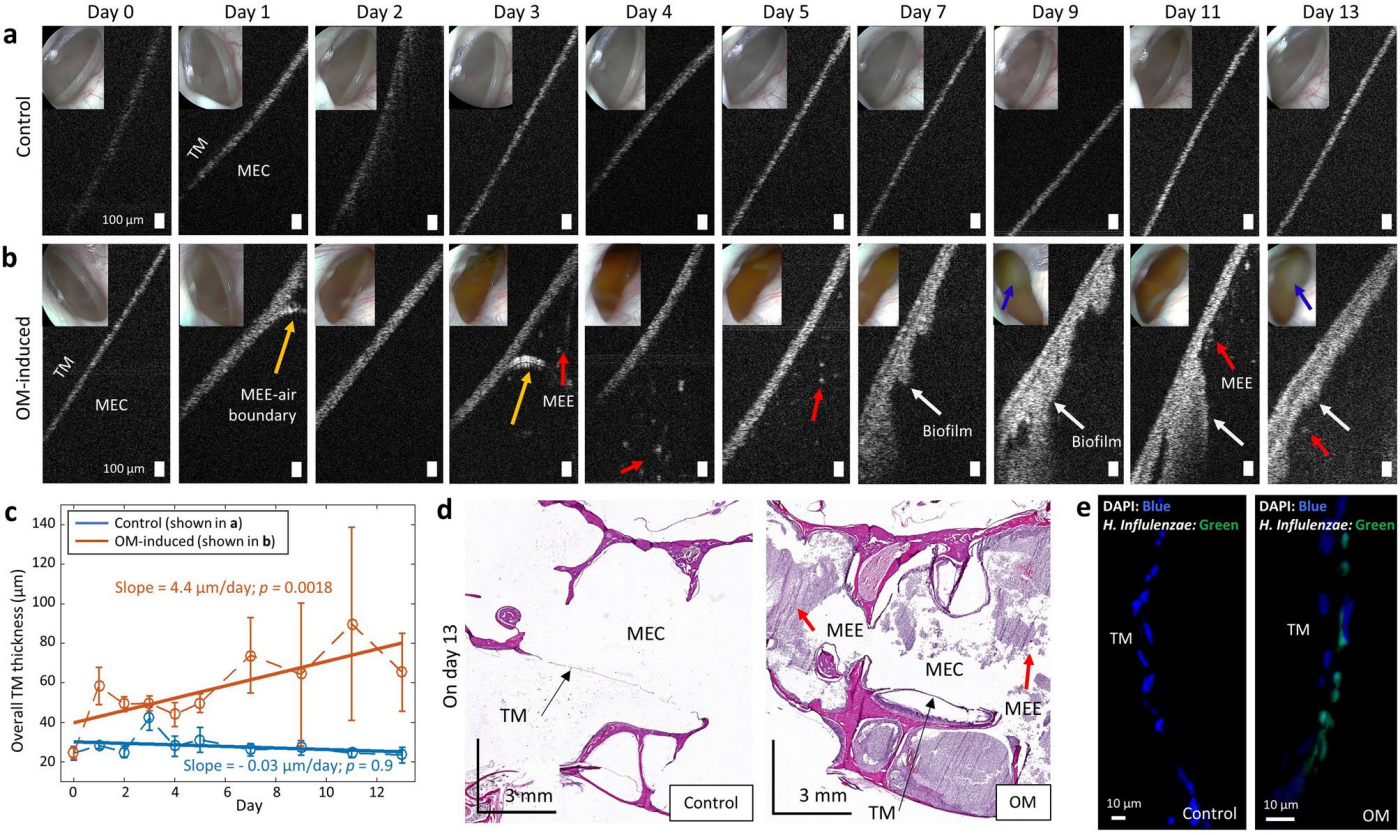
OM-induced changes in the middle ear cavity detected using OCT. Representative OCT and otoscopy (inset) images of (**a**) control chinchilla without induced AOM, and (**b**) AOM-induced chinchilla during the progression of OM. Yellow arrows indicate a MEE-air boundary, red arrows show scattering particle aggregates suspended in the MEEs, and white arrows indicate biofilm. Blue arrows in otoscopy images indicate white biomass behind the TM. (**c**) Plot of the averaged TM thickness measured from OCT with standard deviation for a specific animal shown in (**a**,**b**). The solid line was determined from the linear polynomial fitting. The corresponding p-value on the fitted slope was determined from a two-sided *t* test with the null hypothesis of a slope of zero. A positive slope with a p-value less than 0.05 indicates a significant increasing trend in the thickness. (**d**) Histologic images on day 13 demonstrate distinct differences in the middle ear cavity (MEC) where significant amount of MEE and immune cell infiltration were present in OM-affected bulla, but absent in the control without induced AOM. (**e**) Confocal laser scanning microscope images from fluorescence in situ hybridization (FISH). A nuclei stain (DAPI) detects unspecified genetic components, whereas the green fluorescence indicates the presence of *Haemophilus influenzae* on the TM. Scale bars in OCT, histologic images, and FISH represent 100 µm, 3 mm, and 10 µm, respectively.

Representative OCT images of a control middle ear and an AOM-induced middle ear are shown in Fig. 2. The control chinchilla without induced AOM, Fig. 2a, showed a straight, angled, and thin TM, consistently through day 13 without a significant difference in the thickness. The thickness of the control TM was around 20–30 µm, depending on the region of the TM. In the OM-induced chinchilla, the cross-sectional OCT image revealed a MEE-air boundary (yellow arrows) suggesting the presence of MEE on day 1 after inoculation (Fig. 2b).

On day 4 of Fig. 2b, particulate-like scatterers (red arrows) in a dark low-scattering background was shown. In OCT, a dark background indicates a low amount of light scattering, such as the air in the ear canal above the TM (upper left regions of OCT images). When diffuse scattering particles are present against a dark background, this suggests dilute scattering particles in a clear liquid, such as a MEE with greater water content. On day 7 of Fig. 2b, an additional layer attached to the TM was visualized (white arrows), with an inconsistent thickness. This additional layer appears brighter or more highly scattering in OCT, indicating that the layer was composed of the denser, tissue-like clusters, such as biofilm. The presence of this layer partially corresponded to the bright highly-scattering biomass observed behind the TM in the otoscope images (blue arrows), which was previously confirmed as the biofilm^30^.

Until day 13, this additional layer continued to accumulate, which showed a statistically significant increasing trend over time periods (a linear slope of 4.4 µm/day, with *p* = 0.0018, in Fig. 2c). Note that a 1st degree linear regression model was used to determine a slope and an intercept of the linear model, and a two-sided *t *test was performed to determine if the slope is significantly different from zero. The null hypothesis was that the slope of the linear regression is zero, which indicates no trend with random fluctuations. When the *p-*value was less than 0.05, we concluded that there exists a significant trend of thickness over time. The positive slope of the model indicates an increasing trend, while the negative slope of the model denotes a decreasing trend. From our previous study, we validated with FISH that the additional structure adhered to the human TM (detected by OCT) during OM was biofilm^10^. The presence of a fully filled MEE was identified in the endpoint histologic findings, in Fig. 2d, and the presence of biofilm was validated with FISH in Fig. 2e. Furthermore, due to its high imaging speed, OCT could show varying fluid levels between cross-sectional images, providing three-dimensional information about the volume of fluid inside the middle ear cavity. This was demonstrated in Supplementary Movie S1 online. When there was a scant amount of MEE, the boundary between the MEE and the air was visualized in OCT, which corresponded to the fluid bubbles in the otoscope images (see Supplementary Movie S1 online).

**Figure 3:**
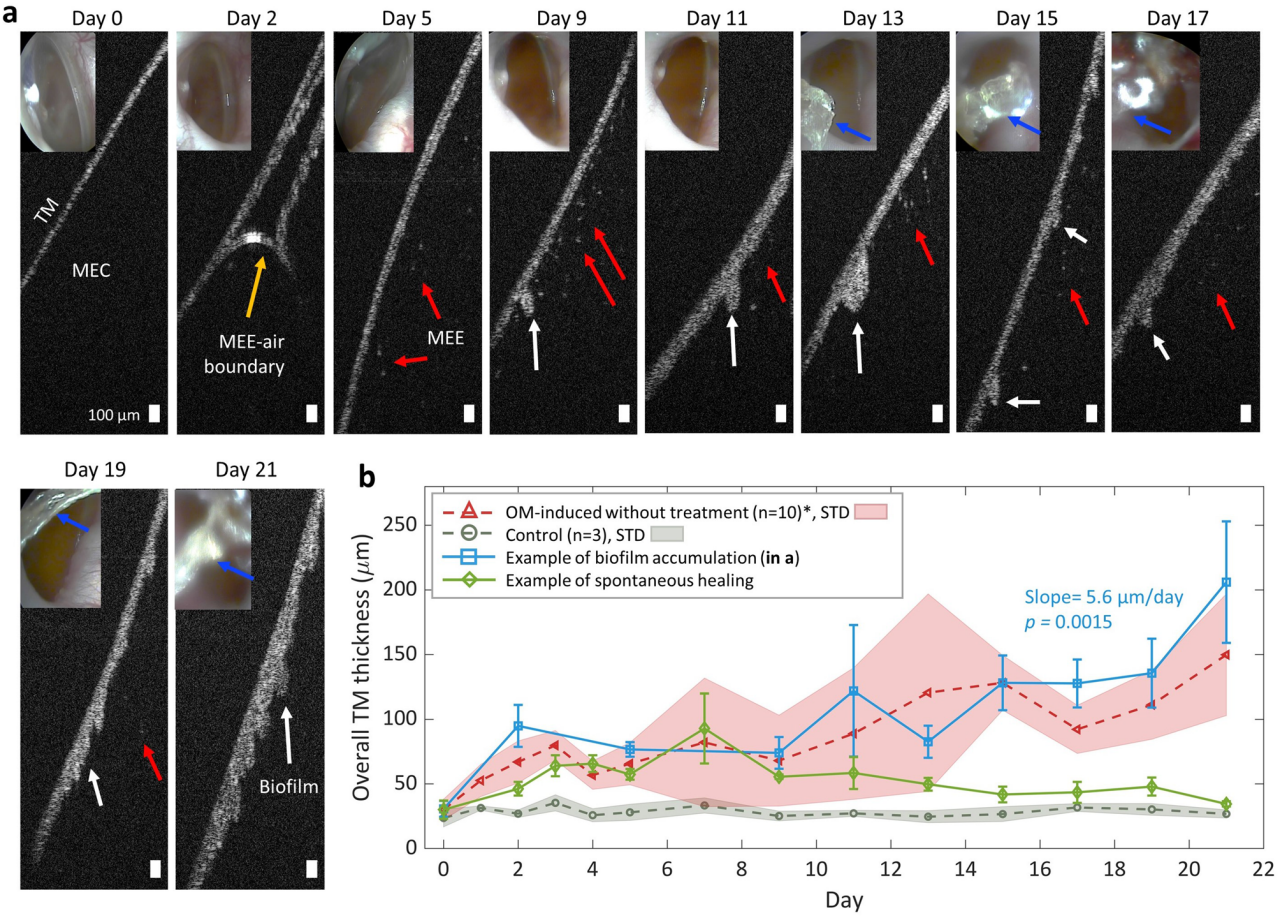
Formation and growth of a TM-adherent biofilm in vivo. (**a**) OCT and otoscopy (inset) images showing the formation of the biofilm in an AOM-induced chinchilla. Starting day 9, a cluster of biomass adhered to the TM was observed, which continued to accumulate until day 21 (white arrows). Blue arrows indicate earwax that limited visual assessment of the TM. Scale bars represent 100 µm. (**b**) The averaged TM thickness and standard deviation were plotted for the control (n = 3) and OM-induced group (n = 10), where n indicates the number of animals. Note that animals longitudinally tracked for longer than day 3 post-inoculation are included in the plot. The thickness measured from the specific animal in (**a**) is shown in blue. The positive slope from the linear polynomial fitting with a *p-*value of 0.0015 indicates a significant increasing trend in the thickness. *STD* standard deviation.

The high-resolution cross-sectional imaging capability of OCT enabled the visualization of very thin amounts of biofilm, where the white biomass was not visible by standard otoscopy. In Fig. 3, the baseline image on day 0 prior to OM induction showed a normal TM. The presence of MEE with particulate-like scatterers was visualized from day 2 through day 21 (red arrows). On day 9, a cluster (white arrow in Fig. 3a) adherent to the TM started to accumulate until day 21, when “mushroom”-like extensions were observed. These structures are characteristic of later-stage biofilms, and break off to form planktonic aggregates of biomass that will distribute and settle on other surfaces to propagate. The overall thickness of the TM and adherent biofilm showed a substantial increase from ~ 31 µm on day 0 to ~ 210 µm on day 21. This potentially demonstrates how the biofilm adhered to the TM and developed over the course of the infection. The averaged TM thickness from the control and OM-induced group is shown in Fig. 3b, and the TM thickness from the specific animal shown in Fig. 3a is shown in blue. For comparison, an example of a spontaneously healed animal after bacterial inoculation is shown in green. The statistical analysis showed a significant increase of TM thickness across the infection period in the OM-induced group. Furthermore, it is important to note that the TM in standard otoscopy was hardly visualized on day 15, 17, and 21, due to the presence of earwax (blue arrows). However, OCT could still generate cross-sectional views inside the middle ear by targeting the narrow light beam toward the partial view of the TM.

In general, the presence of the MEE was identified starting on day 1–2, as verified from OCT images, from 15 out of 16 (94%) AOM-induced chinchillas. The presence of the MEE was determined when additional scattering particles from a MEE or a MEE-air boundary were visualized in any of the acquired OCT images. On the other hand, a clear middle ear was determined when no scattering particles within the middle ear cavity were visualized, along with a smooth TM, in all of the OCT images acquired. The thickness of the TM increased as OM developed due to inflammation and potential biofilm adhered to the TM, while the thickness of the TM remained statistically the same for the control group. The presence of the biofilm was visualized starting on day 5–7 from OCT images obtained from 10 out of 16 (63%) AOM-induced chinchillas. Interestingly, 2 AOM-induced chinchillas (13%) started to heal naturally on day 13 after inoculation (example shown in Fig. 3b). This was determined by the absence of MEE and biofilm from the OCT images. A summary of longitudinally tracking the OM progression is shown in Table 1.

**Table 1:** Summary of longitudinal tracking of experimental OM in chinchilla.

	Conditions at the end time point^a^			
	Clear ears (%)	Ears with biofilm (%)	Ears with MEE (%)	Ears with MEE and biofilm (%)
Control without induced AOM (n = 4, ears = 8)	8 (100%)	–	–	–
NTHi-infected without treatment (n = 16, ears = 31)	4 (13%)	–	11 (35%)	16 (52%)

^a^The ears with a complete blockage of the ear canal at the end time point were excluded for this analysis.

**Figure 4:**
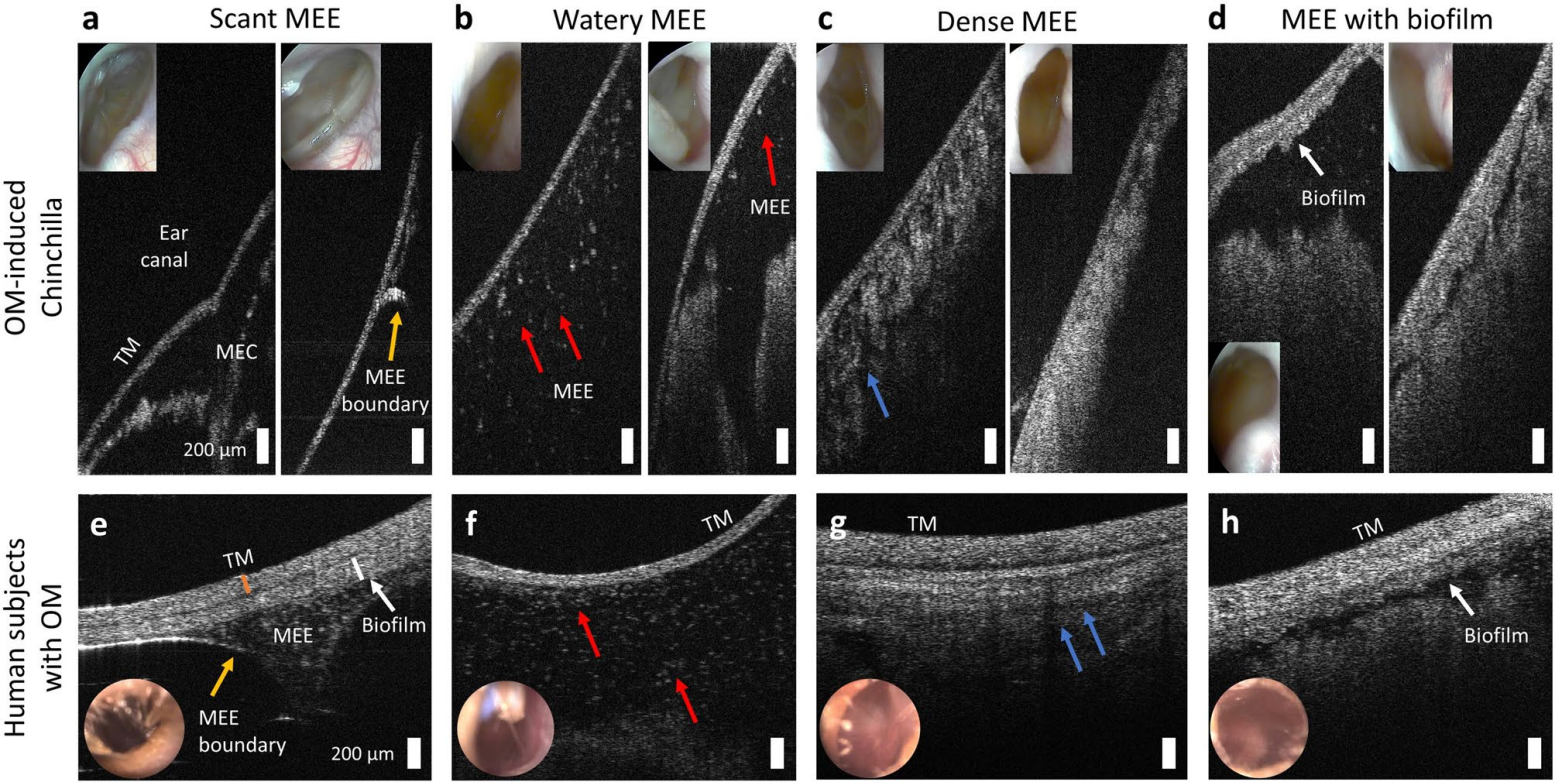
OCT images of different MEE representations in chinchilla compared with those in human subjects. (**a**) Retracted TM and the presence of a scant MEE. **(b)** Particulate-like scatterers present in transparent fluid. (**c**) A dense, highly scattering tissue-like (brighter OCT signals) MEE (blue arrows). (**d**) The presence of a TM biofilm as well as dense biomass. Images were acquired from different chinchillas. (**e**) OCT image of a scant MEE and biofilm from a 3-year-old human subject diagnosed with bilateral recurrent acute suppurative OM. Inset figures represent the otoscope surface view of the TM. (**f**) OCT and otoscope images of a fully filled watery MEE from a 2-year-old subject diagnosed with chronic serous OM. (**g**) OCT and otoscope images from a 1-year-old subject diagnosed with recurrent acute OM. (**h**) OCT and otoscope images of biofilm and MEE from a 14-month-old subject diagnosed with recurrent acute OM. All scale bars represent 200 µm.

Moreover, a wide variability of MEEs in AOM-induced chinchilla was observed from OCT images, as summarized in Fig. 4. OCT was able to capture different middle ear conditions, such as the inflamed and/or thickened TM, the bulging or retracted TM, the middle ear with a scant MEE (Fig. 4a), and different types of MEEs due to varying optical scattering (turbidity). In OCT, the watery MEE (Fig. 4b) was defined as a MEE with particulate-like scatterers in a dark background, and the dense MEE (Fig. 4c) was defined as MEE with whiter/brighter (blue arrows), tissue-like features, which may also contain biofilm. Furthermore, the biofilm adhered to the TM as well as the MEE were observed from advanced stages (Fig. 4d). It is worthwhile to highlight that these features highly resembled in vivo OCT images collected from pediatric subjects with OM, as shown in Fig. 4e–h (see the “OCT images of human subjects with OM” section in “Materials and methods”). This suggests that the OCT findings from chinchilla animal models can be highly translatable to human studies. In practice, the watery MEE can be related to a clinically defined serous MEE, whereas the dense MEE may be close to a clinically defined mucoid MEE.

### Impact of antibiotic treatment on MEE and biofilm

**Figure 5:**
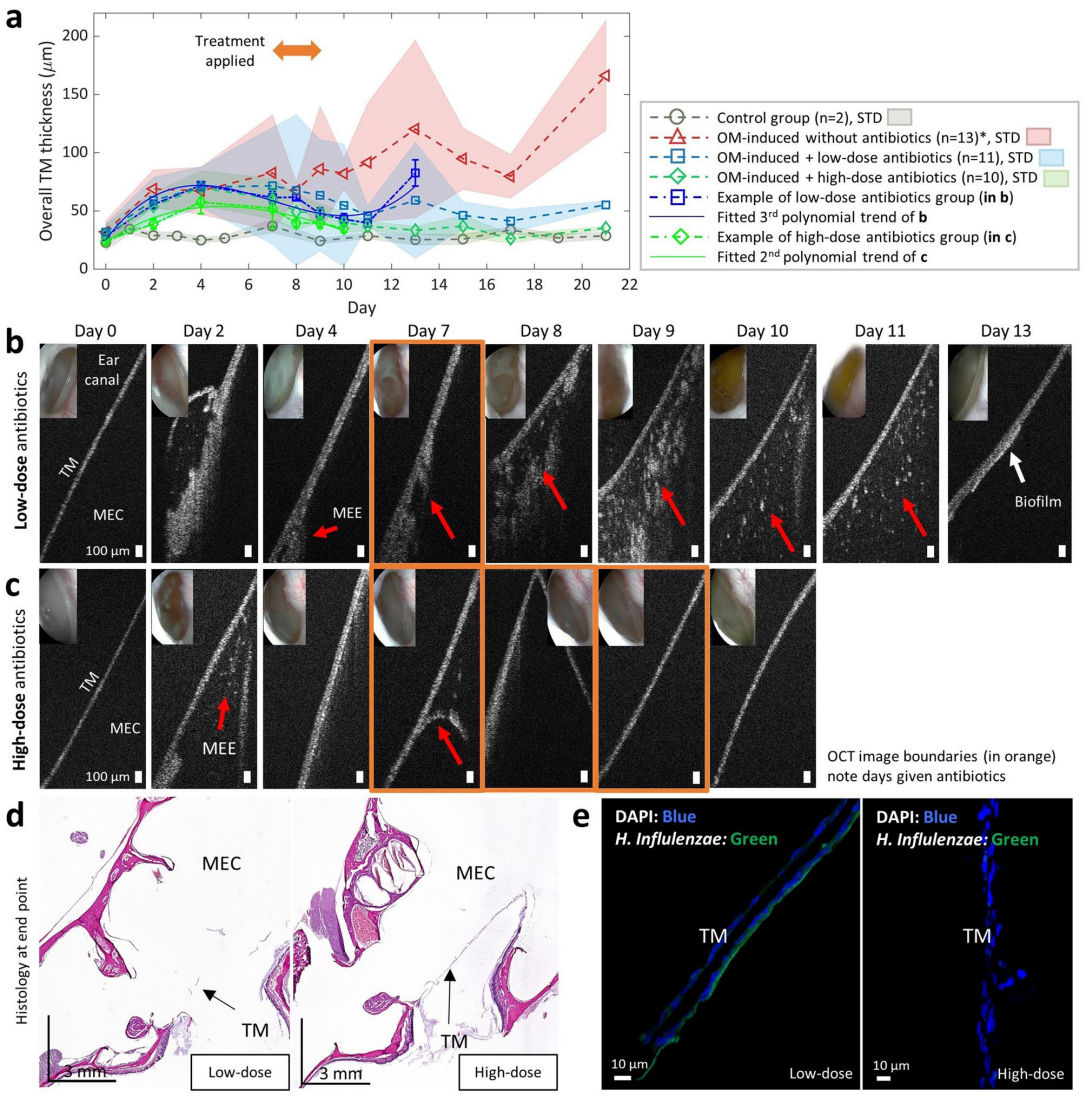
Clearance of MEE after antibiotic therapy longitudinally observed with OCT. (**a**) Plot of the averaged TM thickness and standard deviation in the control, OM-induced without antibiotics, OM-induced with low-dose and high-dose antibiotics groups, where n indicates the number of animals. Note that animals longitudinally tracked for longer than day 3 post-inoculation are included in the plot. Plots from the representative animals in (**b**) and (**c**) are shown in solid blue and green lines, respectively. OCT and otoscopy (inset) images of AOM-induced chinchillas in (**b**) low-dose and (**c**) high-dose antibiotic treatment groups. In (**b**), OCT revealed the absence of MEE and the presence of an additional scattering layer affixed to the TM (white arrow) on day 13. In (**c**), a chinchilla receiving high-dose treatment displayed resolution of OM with no MEE detected on and after day 9. Scale bars in OCT represent 100 µm. (**d**) Histologic images of (**b**) and (**c**) on day 13 and day 10, respectively. (**e**) Confocal laser scanning microscope images from fluorescence in situ hybridization (FISH). A nuclei stain (DAPI) detects unspecified genetic components, whereas the green fluorescence indicates the presence of NTHi on the TM. *Animals in the OM-induced without antibiotics group from Fig. 3b are also included. Three animals in the infected without antibiotics group spontaneously healed and were not included in the plot in (**a**). *STD* standard deviation.

Next, the effects of antibiotic treatment on AOM-induced chinchilla were investigated with a total of 32 chinchillas (control n = 2 and infected n = 30). Among 30 infected chinchillas, 7 chinchillas had no antibiotic treatment, whereas 12 and 11 chinchillas received low-dose and the high-dose ceftriaxone, respectively (see the “Experimental OM” section in “Materials and methods”). The averaged TM thickness with standard deviation in different groups is plotted in Fig. 5a. Representative sets of OCT images showing the clearance of the MEE after the antibiotic treatment are shown in Fig. 5b,c. On day 2 of Fig. 5b, the TM thickness increased to around 56 µm, indicating the inflammation on the TM but without an obvious presence of MEE. On day 4, the presence of a watery MEE was visualized in OCT. After antibiotic treatment on day 7, the presence of MEE was detected by OCT until day 11. From day 8 to day 11, the transition from a dense MEE to a watery MEE type was noticed. On day 13, the MEE was absent, but a thin scattering layer remained attached to the TM (white arrow), indicating the presence of a potential biofilm. Additionally, another chinchilla in the high-dose treatment group (Fig. 5c) showed the clearance of MEE after three consecutive treatments.

In Fig. 5a, the thickness from the specific animals shown in Fig. 5b,c is plotted in solid blue and green lines, respectively. For the animal shown in Fig. 5b, a cubic polynomial function fitted well to the changes in the TM thickness over time (R^2^ = 0.89), and the fitted coefficients are all significantly different from zero, which confirms the curvature of the trend. The TM thickness was increased at first (day 4), sequentially dropped after the antibiotic treatment on day 7, and increased again on day 13 for the chinchilla in the low-dose ceftriaxone group. Due to the acute inflammation in the TM during the first few days of inoculation, the thickness increased and was high around day 3–4 post-inoculation for some animals, and then decreased. However, this trend was not observed from all animals, as shown in the averaged thickness trend of the OM-induced group in Fig. 3b and Fig. 5a. Note that an increase in the thickness on day 13 is due to the thin TM biofilm (white arrow in Fig. 5b). Similarly, a quadratic polynomial function fitted well to the TM thickness changes in the chinchilla shown in Fig. 5c from the high-dose ceftriaxone group (R^2^ = 0.85), and the fitted coefficients are all significantly different from zero.

The histology image (Fig. 5d) of the chinchilla in the low-dose ceftriaxone group contained evidence of inflammation, while that of the chinchilla in the high-dose ceftriaxone group showed a clear middle ear cavity at the end point. Furthermore, the presence of bacteria adhered to the TM in the low-dose group was confirmed with FISH (Fig. 5e), suggesting the great potential of OCT for detecting subtle amounts of biofilm that may remain after antibiotic treatment. In comparison, note that the otoscopy images showed clear TMs at the end point for both animals. These results demonstrate the high sensitivity of OCT in detecting micron-scale structures behind the TM, subtle changes in the TM thickness, and subtle scattering and density differences in MEEs, which was not possible from current non-invasive measurement techniques.

**Figure 6:**
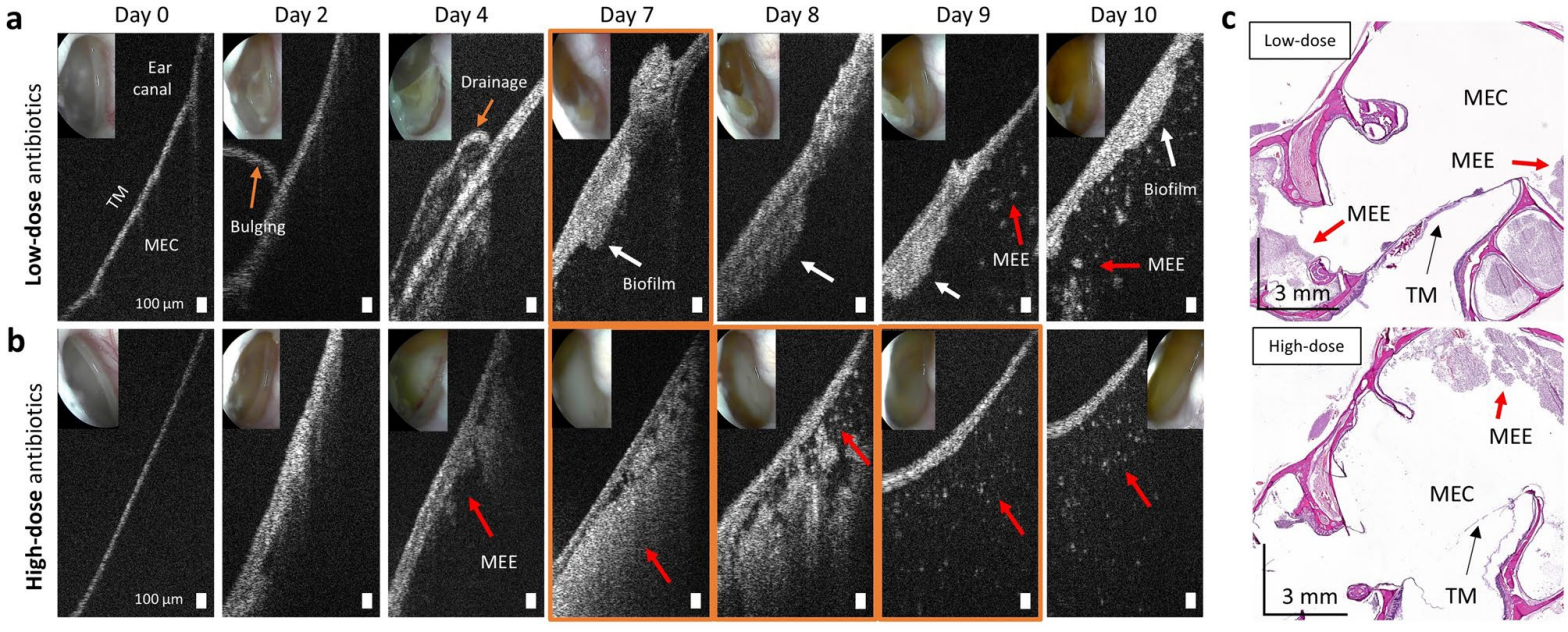
The presence of MEE and biofilm remained after antibiotic therapy. OCT and otoscopy (inset) images of two AOM-induced chinchillas from (**a**) low-dose and (**b**) high-dose antibiotic treatment groups. Both chinchillas showed the presence of the MEE (red arrows) through day 10, and a thick biofilm was visualized (white arrows). OCT image boundaries in orange note days given antibiotics. Scale bars represent 100 µm. (**c**) Histologic images of (**a**) and (**b**) on day 10, visualizing the MEEs in the middle ear cavities (MECs).

On the other hand, representative results showing the presence of the MEE despite the antibiotic treatment are described in Fig. 6. In the chinchillas receiving low-dose ceftriaxone, Fig. 6a, the purulent drainage and the MEE (red arrow) was observed with OCT on day 4. On day 10, the presence of the additional layer with inconsistent thickness was obvious, indicating a potential biofilm (white arrows). It is worthwhile to highlight that OCT detected comparable spatial features (purulent drainage) of the TM between different days, emphasizing its capability as a reliable longitudinal tracking tool. The other bulla from the high-dose treatment group (Fig. 6b) also contained the MEE through day 10. However, it is noteworthy to mention that the MEE was transitioning from a dense MEE (day 7) to a watery MEE (day 9) after the antibiotic treatment, similar to the demonstration in Fig. 5b. This suggests that this chinchilla may be in the process of MEE resolution. The histology findings (Fig. 6c) from both animals on day 10 showed the presence of the MEE. An opposite example of a chinchilla not responding to the antibiotic treatment is shown in Supplementary Fig. S1 online, highlighting much fewer changes in the optical characteristics of the MEE and biofilm even until day 17.

The summarized results of tracking the antibiotic treatment response are described in Table 2. This was determined solely based on the middle ear conditions at the end time point. Two out of 7 chinchillas in the infected without treatment group had complete resolution of biofilm and MEE. As hypothesized, an increased number of chinchillas showed the clear middle ear with the higher dose of antibiotic treatment. However, each animal had a differential response to the treatment regimen, which is similar to responses noted in humans, emphasizing the reliability of this animal model.

**Table 2:** Summary of antibiotic treatment response.

	Conditions at the end time point^a^			
	Clear ears (%)	Ears with MEE (%)	Ears with biofilm (%)	Ears with MEE and biofilm (%)
Control without induced AOM (n = 2, ears = 4)	4 (100%)	–	–	–
NTHi-infected without treatment (n = 6, ears = 12)	3 (25%)	5 (42%)	–	4 (33%)
NTHi-infected with low-dose ceftriaxone (n = 12, ears = 21)	7 (33%)	6 (29%)	1 (5%)	7 (33%)
NTHi-infected with high-dose ceftriaxone (n = 11, ears = 21)	9 (43%)	8 (38%)	–	5 (24%)

^a^The ears with a complete blockage of the ear canal at the end time point were excluded for this analysis.

## Discussion

Biofilm formation has been identified as an important hallmark of OM and is an important mechanism for bacterial persistence and survival during OM^31^. To validate and assess the biofilm in chinchilla, commonly used imaging modalities including FISH, confocal scanning laser microscopy (CSLM), scanning electron microscopy (SEM), and immunohistochemical techniques, all require euthanasia of animals, such as via pentobarbital-based euthanasia solution. Thus, the ability to quickly, non-invasively, and longitudinally visualize middle ear biofilms with OCT is highly advantageous for various OM studies and diagnostics in humans.

OCT provides non-invasive, high-speed, depth-resolved images with cellular resolution. As such, it is an ideal tool for longitudinal and translational studies. The development of the handheld OCT probe is critical for in vivo middle ear imaging, and many studies have shown the promise of OCT as an advanced technique to improve current diagnostic tools in humans^25,32,33^. The quantified TM thickness from OCT has been investigated to differentiate types of OM^29^, and the high speed and resolution of OCT can measure the displacement of the TM in pneumatic otoscopy^34^. OCT has been used to estimate viscosity of MEEs^35^ and categorize relative fluid level and types of MEEs^24^. More recently, a machine learning platform has been developed for middle ear OCT images to classify middle ear conditions^36^.

In this study, OCT was employed to assess the middle ear cavity in AOM-induced chinchillas in vivo. The micron resolution of the images provided quantitative information in overall thickness of the TM as well as the presence of bacterial biofilm adhered to the TM throughout the course of the infection. One study reported the TM thickness of 15 µm and 26 µm for the control and AOM-induced chinchilla, respectively, based on a histology section^37^. Non-destructive OCT measurement of the TM from this current study showed slightly greater values, with additional information on potential TM-adherent biofilm. Furthermore, different types and amounts of MEEs were visualized with OCT, which partially agreed with the features from standard otoscopy, such as air bubbles, TM opacity, and white biomass. These differences, however, did not always correlate with the time course of the disease, highlighting different innate immune responses in each chinchilla. The scattering properties in the MEE significantly changed over the course of the OM development as well as during the process of MEE resolution. In addition, although these types of MEEs vary between animals and the imaging day, the optical properties of a MEE in bilateral ears exhibit high similarities, as expected from the bilateral inoculation. Indeed, the OCT images of MEEs and the biofilms from chinchillas showed high similarities with those collected from pediatric subjects with OM, as shown in Fig. 4.

OCT provides superior information compared to otoscopy, the standard diagnostic method to monitor middle ear conditions. OCT has a greater sensitivity for detecting even small amounts of MEE as well as thin biofilm layers affixed to the TM. A capability to estimate the amount and the type of MEE is also advantageous when monitoring the treatment and the development of OM, especially for therapeutic studies. Furthermore, OCT can generate images inside the middle ear even with earwax or discharge present, as long as the TM is located within the imaging depth and the TM is not completely blocked.

The results of this study demonstrated a statistical decrease in the overall TM thickness and the resolution of MEEs from animals with known AOM which were treated with antibiotics. This was an expected outcome. Much of the current philosophy around considering “watchful waiting” or delayed antibiotics in children with presumed AOM is related to the fact that the diagnosis can be difficult to make with current otoscopic techniques, that the middle ear is not well assessed, and that there is some natural resolution of AOM in children related to the patient’s own immune system’s ability to limit the disease. However, it is also known that for children in whom an accurate diagnosis of AOM can be made, and an appropriate antibiotic prescribed, these children will have fewer complications and fewer longer-term difficulties with their AOM^38^. In these experiments with controlled conditions we were able to demonstrate the benefit of OCT in specific animals within groups. Much like the human condition of AOM, this study had animals in the non-antibiotic group that had spontaneous resolution of their AOM. In some of their OCT images, it was possible to determine a relatively less severe infection and less amount of effusion.

Translating these findings to a human population, one could envision using OCT in a clinical setting, and with the added benefit of in-depth visualization of the TM and middle ear space, a clinician could reliably decide which patients may benefit from avoiding antibiotic treatment, which has its own difficulties related to side effects and eventual development of antibiotic resistance. In addition, in the treatment groups, OCT provided novel information not currently available using otoscopy to predict which chinchillas were progressing toward disease resolution. In longitudinal imaging, it was possible to demonstrate resolution of biofilms, lessening of TM thickness, and changes in the overall consistency of the effusion—all suggesting progress toward resolution of the AOM. One can also envision a clinician utilizing such information to inform decisions on the need for longer or shorter courses of antibiotics, or the necessity to change antibiotic regimens, or simply advise parents on the natural course of their child’s illness, as information such as child behavior, sleeping patterns, and diet are often part of the equation in trying to decide next steps in a child with AOM.

One major complexity in a longitudinal human study is the poor follow-up rate after the initial clinic visit and treatment. If patients come back for follow-up, they often do not preschedule their appointment or return to the same clinic, often going to convenient care options or in the case of more urgent conditions, to the emergency room. However, prescheduling appointments at predetermined locations is necessary so that an OCT system can be sited and ready to use. Furthermore, each patient will have OM caused by different bacterial strains and/or viruses, which will lead to varying middle ear responses. As a result, hundreds of human subjects will be needed. On the other hand, this study with a chinchilla animal model provides us a control for longitudinal tracking and response. Note that human imaging is likely less complex to perform than imaging chinchilla animal models, but it is the scheduling and current healthcare system logistics that present a challenge for conducting a longitudinal study in the human population. This can be overcome with a larger effort, such as an involvement of more physicians, more available OCT systems and researchers, more patients, and a longer study duration. If follow-up imaging intervals before and after the treatment can be maintained between subjects, the treatment response of middle ear conditions can be precisely evaluated and compared with OCT.

There are a few intrinsic challenges of OCT for imaging the middle ear cavity. One inherent challenge as an optical imaging technique is the limited imaging depth. Thus, OCT images provide information for the most lateral portion of the middle ear cavity but, with current technology, the images do not penetrate to the bottom of the middle ear cavity (the promontory). Another difficulty is that standard OCT is purely an anatomical imaging technique based on spatial variations of the refractive index, which cannot provide functional descriptions of the tissue. There also exist many design considerations when constructing the OCT system for the middle ear, such as having strong white-light illumination to visually guide the OCT imaging beam, and implementing disposable otoscope tips. More details in the system requirements can be found in Monroy et al. and MacDougall et al.^20,39^.

Several additional challenges exist for imaging chinchilla middle ears with OCT. First, the accessibility of the middle ear is more limited in chinchillas than in humans. The ear canal of the chinchilla is generally narrower and more angled than those of humans. Thus, the form factor of either an endoscope or a small handheld probe tip is required for OCT imaging system to direct the beam into the small, tortuous ear canal of chinchillas. Second, the TM is more highly angled to the ear canal, which can cause image artifacts (flipped image in OCT). Third, the TM thickness in chinchillas (15–30 µm) is thinner than that in humans (70–120 µm), which may require a superior axial resolution (much less than 10 µm) of the OCT imaging system to resolve smaller features within the chinchilla TM. Lastly, the correction of refractive index error is required for quantitative assessment, since an incident beam angle to the TM surface can be around 40°–70°.

There exist a few limitations in the study. First, the cross-sectional images were not co-registered between different imaging days. Acquiring volumetric OCT images to ensure more appropriate longitudinal tracking is ideal for future investigations. Second, the conditions throughout the entire middle ear cavity could not be determined from the OCT images due to its limited imaging depth. This could be more problematic in chinchilla than in human, because of its larger middle ear cavity. In this study, histology at the end time point was necessary to correlate and confirm the middle ear conditions and the findings from OCT. Next, there were a few incomplete datasets, due to the complete blockage of the view of the TM from cerumen (earwax), and/or blood. When OCT imaging was performed daily or every other day during the study, some animals had an inflamed external ear canal. This can be prevented by increasing the imaging interval in future studies. In addition, since the clearance of the MEE was determined based on the middle ear conditions at the end time point, chinchillas in the process of MEE resolution at the end time point were not considered to have been successfully treated. Developing adjustable experimental plans based on the OCT images after antibiotic treatment will help observe the complete course of the antibiotic treatment response. Lastly, the intrinsic limitations as a chinchilla study remain, despite this model being the most representative of OM in humans.

As OCT can non-invasively visualize the in vivo response of middle ear biofilm and MEEs, studying the impact of antibiotic treatment in conjunction with OCT may be a promising way to better understand the therapeutic responses in chinchilla OM models as well as in patients. As the next step, a longitudinal study with a large number of patients receiving antibiotic treatment is necessary to statistically characterize drug responses, which can ultimately help develop an automated algorithm to examine treatment efficacy.

## Materials and methods

### Optical coherence tomography system

A portable, handheld spectral-domain OCT system with a superluminescent diode (Superlum) centered at 860 nm with a bandwidth of 130 nm was developed. The line scan camera-based spectrometer (Wasatch Photonics) at 32 kHz A-scan rate was used with a MEMS scanning unit (Mirrorcle Technologies). This system enabled an imaging depth of around 3 mm, a field-of-view of 4 mm, and a depth resolution of around 4.5 µm. Each OCT image was displayed in real-time and collected at a rate of 20 frames per second. Simultaneous camera imaging (XIMEA) in addition to OCT was performed to visualize the TM surface and to provide guidance while collecting OCT images. Further details can be found in an earlier publication^29^. In this study, non-OCT expert users operated and acquired the images, emphasizing the ease-of-use of the custom-built system. Around 200 OCT cross-sectional images from each ear were software-triggered and collected during each imaging session. All acquired OCT images were considered to determine the presence of a MEE and biofilm, and the overall middle ear conditions.

### OCT image analysis

Although cross-sectional OCT images provided the depth-resolved view inside the middle ear cavity, quantitative assessment was vital for longitudinal tracking and statistical analysis. In order to quantify the TM thickness, the images scanned around the same region of interest between days, determined from the similarities of the structures, were selected. Images with bad focus of light and artifacts were not used for the analysis. The good image acquisition rate depended highly on the individual ear canal and ear conditions, ranging from 50 to 80%, when the ear canal was not blocked. First, the image was segmented to select the overall TM thickness, which included any biofilm that may have been present and attached to the TM. The segmentation process included median filtering and conversion to binary images with thresholding. Next, the orthogonal thickness was computed (Fig. 1). Since chinchilla TMs in OCT images were steeply angled, the orthogonal thickness was corrected for a refractive index (RI) error by computing the incident angle on the TM, assuming the RI of 1.44^40^. The thickness from around 100 consecutive points in each image was used to calculate the overall TM thickness.

### Bacterial culture

NTHi strain 86028NP was used for the study because of its accessible and complete genomic sequence, as well as baseline data for biofilm formation and infection models^41,42^. The bacteria were grown at 37 °C in 5% CO_2_ in brain heart infusion (BHI) medium supplemented with hemin and NAD, referred to as supplemented BHI (sBHI). The NTHi cultures grown overnight on agar plates were harvested and re-suspended in phosphate buffer saline (PBS) to an optical density at 600 nm (OD600) of 0.15 (~ 10^8^ CFU per ml). The bacteria were serially diluted in PBS to the desired concentration (~ 10^5^ CFU per 0.1 ml per middle ear) for chinchilla middle ear inoculation. The actual concentration of bacterial inoculum was confirmed by plating.

### Experimentally induced OM

#### Part 1: Tracking the formation of MEE and biofilm in chinchilla

AOM-induced chinchillas (n = 16) and control chinchillas without induced AOM (n = 4) were imaged with OCT on selected days after inoculation. All animals were handled according to a protocol (AUA0176) approved by the Institutional Animal Care and Use Committee (IACUC) at the Medical College of Wisconsin. Chinchillas age 6–10 months old (body weight 400–500 g) with mixed gender were purchased and allowed to acclimatize to the vivarium for 5–7 days prior to the middle ear inoculation. All animals entering the study showed no visible signs of illness or ear disease by otoscopic exam. OCT images of TMs were taken prior to inoculation as a baseline for measurement in each animal under anesthesia. Isoflurane, as high as 5%, was delivered to a transparent induction chamber where animals were placed in to anesthetize the animals. The animals were inoculated with NTHi (~ 10^5^ CFU/ear) suspended in PBS (pH of 7.4) via transbullar injection on experimental day 0. All animals were monitored by otoscopic exam and OCT imaging daily for the first 5 days post inoculation, then every other day through the course of the experiment. The imaging time point for Part 1 is shown in Supplementary Table S1 online.

#### Part 2: Tracking the effects of antibiotics on MEE and biofilm

A total of 32 chinchillas were divided into four groups: control chinchillas without induced AOM (n = 2), NTHi-infected without antibiotic treatment (n = 7), NTHi-infected with higher dose (50 mg/kg three times daily) ceftriaxone (n = 11), and NTHi-infected with lower dose (50 mg/kg once daily) ceftriaxone (n = 12). Similar to Part 1, the acclimatized chinchillas were inoculated with NTHi (~ 10^5^ CFU/ear) via transbullar injection on experimental day 0. All animals were monitored by otoscopic exam and OCT imaging every other day for the first 7 days post inoculation. Since OCT was able to detect the TM surface biofilm on day 5–7 post inoculation from the Part 1 experiment, antibiotic ceftriaxone treatment was given starting on day 7 post inoculation. The imaging time point for Part 2 is summarized in Supplementary Table S2 online. For both Part 1 and Part 2, tissue procurement was performed on day 3, 7, 13, and 21 post inoculation with 2–4 chinchillas per time point. Chinchillas were euthanized with pentobarbital-based euthanasia solution via intracardiac injection, while under anesthesia. The bullae were harvested and placed in 10% normal buffered formalin for histologic assessment.

### Histologic assessment

Chinchilla bullae were fixed in 4% paraformaldehyde, decalcified in 10% EDTA pH 7.0 for 14 days, cryo-protected with 20% sucrose in PBS, and embedded in optimal cutting temperature compound for cryosection. The decalcified bullae were serially sectioned at 6 µm and stained with hematoxylin and eosin (H&E). The stained slides were scanned at 0.4 µm/pixel using a TissueScope LE Slide Scanner (Huron Digital Pathology). The scanned images were assessed for the histologic changes in the middle ear cavity.

### Fluorescence in situ hybridization (FISH)

Six-micron cryosections of the middle ear were prepared and detected by FISH using bacterial 16s rRNA probes as previously described^9^. In brief, the slides were dehydrated sequentially with ethanol, then treated with 10 mg/ml lysozyme (Sigma-Aldrich) in 0.1 M Tris-0.05 M EDTA at 37 °C for 1 h, and washed with ultrapure water. Slides were blocked with nonspecific human Cot-1 DNA (Thermo Fisher Scientific) at 37 °C for 6 h. Slides were stained with a 16s rRNA probe P-Hinf (GCCATGATGAGCCCAAGTGG-C3-fluorescein, *H. influenzae*). Slides were mounted with SlowFade Gold antifade reagent with DAPI (Thermo Fisher Scientific) and evaluated using CLSM (LSM 510; Carl Zeiss, Oberkochen, Germany) and LSM Image Browser software (Carl Zeiss).

### Statistical analysis

The mean and standard deviation of the TM thickness over different locations were calculated for each chinchilla at each time point. Then, a linear, quadratic, or cubic curve was fitted over time, depending on the distribution of the data. A two-sided *t *test on the fitted coefficients was performed to determine if the coefficient is significantly different from zero. For a linear model, significant positive and negative slopes indicate increasing and decreasing trend of the TM thickness, respectively. The statistical analysis was performed using Statistical Analysis Software (SAS) 9.4 and MATLAB R2019b.

### OCT images of human subjects with OM (Fig. 4e–h)

A protocol approved by the Institutional Review Boards (IRBs) at the University of Illinois at Urbana-Champaign and Carle Foundation Hospital in Urbana, IL, was used. Written informed consent was obtained from a parent or legal guardian of all pediatric subjects (age ranging from 1 to 3 years old) diagnosed with OM prior to imaging. OCT imaging and standard otoscopy were performed in a standard exam room at Carle Foundation Hospital. A total of around 10 min per subject was spent for the measurements in the clinical environment.

All methods involving humans and chinchillas in this study were carried out in accordance with relevant guidelines and regulations from IRBs and IACUC, respectively. Methods involving chinchillas complied with the ARRIVE guidelines.

## Supplementary Information

Supplementary Video.

Supplementary Information.

## Data Availability

The data that support the findings of this study are available from the corresponding authors upon reasonable request and through a collaborative research agreement.
