# Supplementary material for: Longitudinal optical coherence tomography to visualize the in vivo response of middle ear biofilms to antibiotic therapy
Source: Sci Rep. 2021 Mar 4;11:5176. doi: 10.1038/s41598-021-84543-9 (PMC7933323; doi:10.1038/s41598-021-84543-9)
Supplement: Supplementary file 2 — Supplementary Information. [file 41598_2021_84543_MOESM2_ESM.pdf]

***Supplementary information**

Longitudinal Optical Coherence Tomography to Visualize the *In Vivo* Response of Middle Ear Biofilms to Antibiotic Therapy

Jungeun Won[1,2†], Wenzhou Hong[3†], Pawjai Khampang[3], Darold R. Spillman, Jr.[2], Samuels Marshall[3], Ke Yan[4], Ryan G. Porter[5,6], Michael A. Novak[5,6], Joseph E. Kerschner[3,7*], and Stephen A. Boppart[1,2,6,8*]

[1] Department of Bioengineering, University of Illinois at Urbana-Champaign, Urbana, Illinois.
[2] Beckman Institute for Advanced Science and Technology, University of Illinois at Urbana-Champaign, Urbana, Illinois.
[3] Department of Otolaryngology and Communication Sciences, Medical College of Wisconsin, Milwaukee, Wisconsin.
[4] Section of Quantitative Health Sciences, Department of Pediatrics, Medical College of Wisconsin, Milwaukee, Wisconsin.
[5] Department of Otolaryngology, Carle Foundation Hospital, Urbana, Illinois.
[6] Carle Illinois College of Medicine, University of Illinois at Urbana-Champaign, Champaign, Illinois.
[7] Division of Otolaryngology and Pediatric Otolaryngology, Medical College of Wisconsin, Milwaukee, Wisconsin.
[8] Department of Electrical and Computer Engineering, University of Illinois at Urbana-Champaign, Urbana, Illinois.

† represents co-first author, and * represents co-corresponding author.

Address correspondence to JKerschner@mcw.edu and boppart@illinois.edu.

Supplementary Movie S1. Three-dimensional information that can be used to estimate the volume of fluid inside the middle ear cavity. Top left corner shows a representative otoscopy image of an AOM-induced chinchilla (day 10 post-inoculation).

Supplementary Figure S1. Example of a persistent MEE not responding to antibiotic treatment.

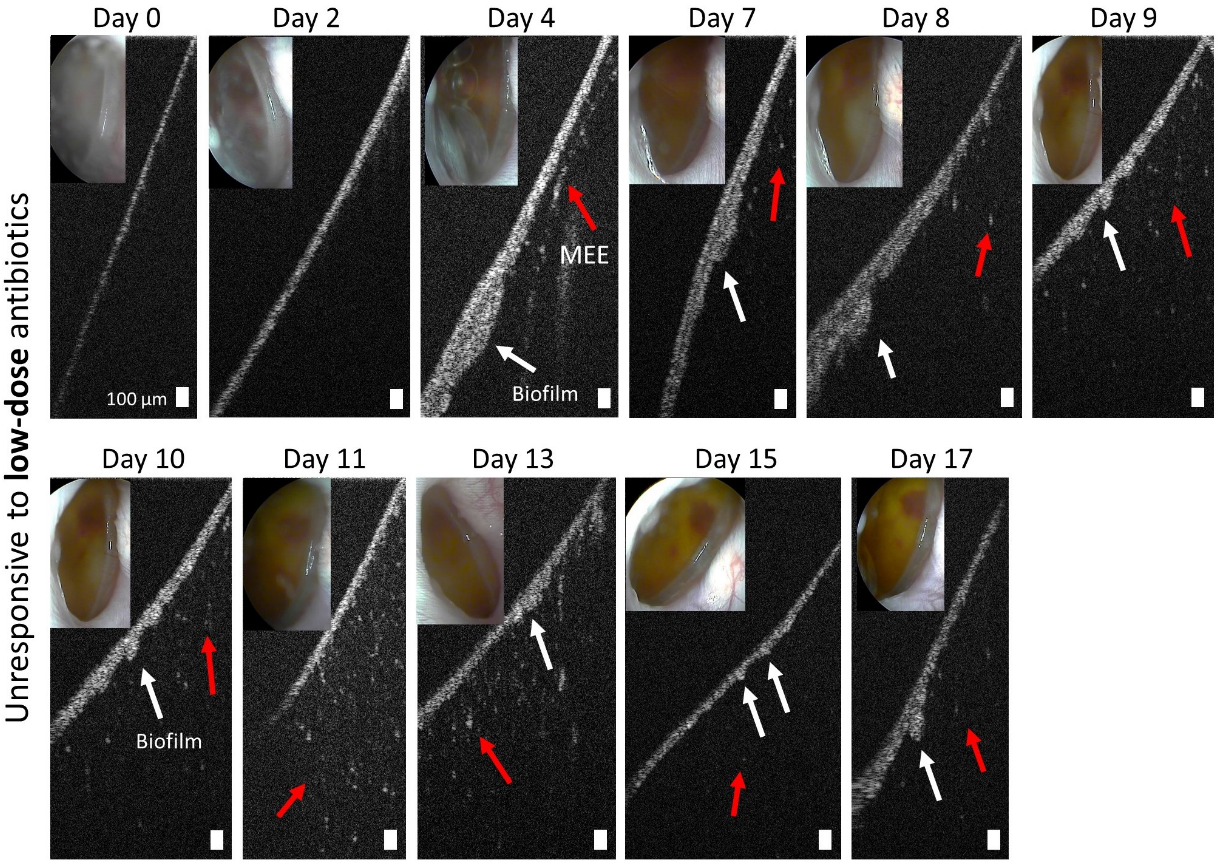

Fig. S1. Example of a persistent MEE not responding to antibiotic treatment. OCT and otoscopy (inset) images show the formation of the MEE (red arrows) and biofilm (white arrows) in an AOM-induced chinchilla. The presence of a thin, TM-adherent biofilm as well as the presence of watery MEE remained until day 17. No significant changes of MEE types were observed, suggesting ineffective antibiotic treatment. Scale bars represent 100 µm.

Supplementary Table S1. Longitudinal imaging time points for tracking MEE and biofilm formation in chinchilla.

Date	Procedure	Imaging data
Day 0	Inoculation; buprenorphine	OCT, otoscopy
Day 1		OCT, otoscopy
Day 2		OCT, otoscopy
Day 3	Buprenorphine	OCT, otoscopy, histology
Day 4		OCT, otoscopy
Day 5		OCT, otoscopy
Day 6	Buprenorphine	
Day 7		OCT, otoscopy, histology
Day 9		OCT, otoscopy
Day 11		OCT, otoscopy
Day 13		OCT, otoscopy, histology
Day 15		OCT, otoscopy
Day 17		OCT, otoscopy
Day 19		OCT, otoscopy
Day 21		OCT, otoscopy, histology

Supplementary Table S2. Longitudinal imaging time points for monitoring the effects of antibiotic treatment on AOM-induced chinchilla.

**Date**	**Procedure**	**Imaging data**
Day 0	Inoculation; buprenorphine	OCT, otoscopy
Day 2		OCT, otoscopy
Day 3	Buprenorphine	OCT, otoscopy
Day 4		OCT, otoscopy
Day 6	Buprenorphine	
Day 7	Antibiotics	OCT, otoscopy
Day 8	Antibiotics<br>(for the high-dose group)	OCT, otoscopy
Day 9	Antibiotics<br>(for the high-dose group)	OCT, otoscopy
Day 10	Ketamine	OCT, otoscopy, histology
Day 11	Euthasol	OCT, otoscopy
Day 13		OCT, otoscopy, histology
Day 15		OCT, otoscopy
Day 17		OCT, otoscopy
Day 23		OCT, otoscopy, histology
